# Cleaning up our disinfectants: usage of antimicrobial biocides in direct-to-consumer products in Australia

**DOI:** 10.1099/acmi.0.000714.v3

**Published:** 2024-02-14

**Authors:** Charles Nunez, Rebecca S. Bamert, Karen Lambert, Francesca L. Short

**Affiliations:** ^1^​ Centre to Impact AMR, Monash University, Clayton, Victoria 3800, Australia; ^2^​ Department of Microbiology, Biomedicine Discovery Institute, Monash University, Clayton, Victoria 3800, Australia; ^3^​ Department of Biochemistry, Biomedicine Discovery Institute, Monash University, Clayton, Victoria 3800, Australia; ^4^​ School of Curriculum, Teaching and Inclusive Education, Monash University, Frankston, Victoria 3199, Australia

**Keywords:** biocide, disinfectant, antiseptic, benzalkonium chloride, quaternary ammonium compound, antimicrobial resistance

## Abstract

In supermarkets and chemists worldwide, consumers are faced with an array of antimicrobial domestic cleaning and personal hygiene products purporting to kill germs and keep people safe. Many of these proven active ingredients (biocides) encourage the development of antimicrobial resistance (AMR) in microbes and microbial populations, in turn increasing the likelihood of AMR infections. In order to understand and address the selective pressure towards AMR posed by the unrestricted use of biocides, it is necessary to understand which biocides are most frequently found in consumer products and the current regulatory framework that governs their use. In this research we survey the biocidal active ingredients in the major categories of cleaning and personal care products available from supermarkets and pharmacies in Australia, and comment on the regulations that dictate how these products are tested and marketed. Benzalkonium chloride and ethanol were the two most prevalent antimicrobial biocides in this study, while triclosan, which is banned in several jurisdictions, was found in a small number of products. In Australia, many antimicrobial consumer products are regulated for efficacy and safety under the Therapeutic Goods Act, but the potential to drive microbial adaptation and AMR is not considered. Overall this survey underscores the broad use and light regulation of antimicrobial biocides in products available to the general public in Australia, and provides an information resource to inform further research and stewardship efforts.

## Data Summary

Source data used for this article is provided as a Supplementary Spreadsheet, available in the online version of this article.

## Introduction

Antimicrobial biocides are compounds that kill or prevent the growth of microbes, but are not used as medicines [1]. Biocides are broadly used as active ingredients in disinfectants and antiseptics in both domestic and healthcare settings [2–4], and are also used as antimicrobial preservatives. In addition to antimicrobial activity, certain biocides have applications as descaling agents or surfactants. Because of their favourable properties, biocides are broadly used in household, toilet and laundry cleaning products and other consumer goods intended for long-term use such as cosmetics and personal hygiene products [5]. Thus, antimicrobial biocides exist in myriad, diverse everyday products, making it difficult for consumers to make informed choices about their use.

Biocide use is a cause for concern due to the potential for microbes to resist the biocides themselves, and the potential for biocide use to contribute to AMR [6]. An estimated 4.95 million deaths were associated with bacterial AMR in 2019 [7]. In Australia alone in 2020, 1031 deaths were directly due to AMR bacterial infections, at a hospital cost of $72 million Australian dollars to treat the five most common AMR pathogens: *Enterococcus* spp., *Escherichia coli*, *Klebsiella pneumoniae*, *Pseudomonas aeruginosa* and *Staphylococcus aureus* [8]. These five pathogens are part of the ESKAPE group, which is known to be the leading cause of nosocomial infections worldwide due to its strong association with AMR [9]. AMR arises primarily from antibiotic use in medical and agricultural contexts [10–12]; as bacteria are exposed to antibiotics, some cells will develop adaptations to withstand the drug. The One Health approach to AMR aims to understand the connections between AMR in humans, animals and the environment [10].

**Fig. 1.**
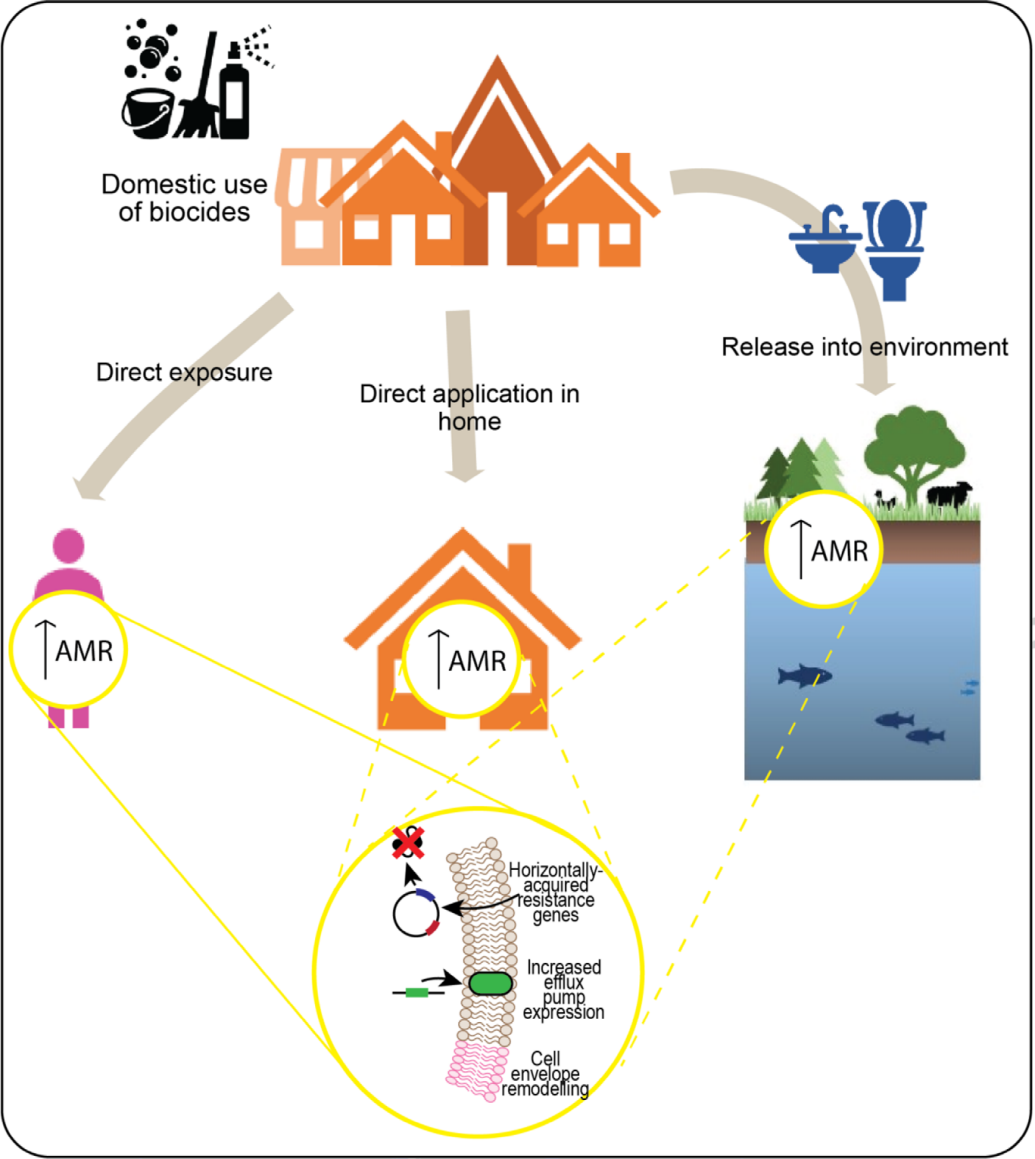
Schematic of exposure due to domestic use of products containing antimicrobial biocides. Microbes associated with humans or the home environment can be exposed to subinhibitory levels of biocide directly (e.g. through incorrect use, expired product, or residual biocide), while environmental microbes can be exposed where biocides are released (e.g. in wastewater). Biocide exposure can increase resistance to antimicrobials through generic mechanisms including increased efflux of chemicals, decreased permeability of the cell wall or membrane, or acquisition of antimicrobial resistance genes.

There is substantial evidence that the use of antimicrobial biocides may unintentionally contribute to AMR [1] (Fig. 1). For example, benzalkonium chloride (BAC), a quaternary ammonium compound (QAC) biocide, promotes the emergence of AMR bacteria [13, 14], notably polymyxin-resistant *P. aeruginosa* [15]. Exposing *E. coli* to a range of biocides resulted in the emergence of cross-resistance, with BAC and chlorhexidine amongst the biocides tested having the greatest impact upon resistance to antibiotics [16]. In *P. aeruginosa*, exposure to the biocide Triclosan resulted in mutants overexpressing the efflux system MexCD-OprJ, resulting in resistance to several antibiotics including tetracycline, ciprofloxacin and erythromycin [17]. Reported biocide-antibiotic cross-resistance is primarily due to the biocides selecting for bacterial mutants with changes to cell-envelope composition or increased efflux pump activity; generic mechanisms which can also increase survival in the presence of antibiotics. In addition, biocides can increase the exchange of AMR-related genetic elements, and can directly antagonize antibiotics in some situations [1, 15, 18, 19]. Adaptation to biocides can occur when bacteria are exposed to them at low, subinhibitory concentrations; not enough to kill the bacteria, but enough to induce a stress response and select for fitter variants. Bacteria can be exposed to low concentrations of biocides in the environment due to the release of these molecules through wastewater streams, and when products are used that have reduced activity due to age or incorrect formulation (Fig. 1). For example, biocides of the quaternary ammonium compound (QAC) class have been detected in hospital wastewater at concentrations that are relevant to *in vitro* AMR development [20, 21]. Determining how much real-world AMR is attributable to biocide exposure is extremely challenging because of the ubiquity of biocide use, the diversity and generality of adaptation mechanisms and the unknowns regarding accumulation of biocides in different environments; these issues have been explored in several recent reviews [1, 22, 23]. However, the strength of laboratory evidence for biocide-antibiotic cross-resistance, and the broad and unrestricted use of biocides, mean that the potential for biocide-driven AMR should be considered when evaluating when, and how, to use products containing these chemicals, particularly in product categories where effective alternatives are available.

In order to understand how biocides may be contributing to the AMR crisis, it is important to know how and in what contexts biocides are being used, particularly in domestic settings where high-level antimicrobial activity may not be necessary. However, there is a lack of systematic information available. Many different biocides are currently on the market in specific consumer products. For instance, sodium hypochlorite, hydrogen peroxide and QACs were among the most frequent biocides in surface and laundry cleaning products in Germany [4], whilst the majority of hand sanitizers are alcohol-based [24]. The potential for biocide-driven AMR has been addressed with regulation in limited cases; notably, in the USA, triclosan and 18 other biocides are no longer permitted in cosmetics and personal hygiene products due to AMR, environment and toxicology concerns [17, 25–28]. The EU Biocidal Products Regulation in Europe employs a similar policy, in which biocides that pose harm to humans, animals or the environment are prohibited as consumer product ingredients [29]. In 2022, Canada proposed a new biocide regulation system that will allow consistent regulation of biocides based on risk, under a single framework [30].

This work aims to provide systematic information on the types of antimicrobial additives biocides present in antimicrobial products designed for home or personal use, in an Australian context. We focus solely on direct-to-consumer products, rather than those for commercial, industrial, agriculture or healthcare use, as their use – particularly following changes in consumer habits following the Sars-CoV-2 pandemic – is likely to account for a large proportion of biocide exposure, and because ingredient information on such products is readily available. The dataset can serve to inform future research and regulation efforts.

## Methods

### Survey of biocides

To investigate hundreds of Australian consumer products efficiently and maximize our coverage, we searched for products available through official websites of Australia’s leading supermarkets and pharmacies (Coles, Woolworths, Chemist Warehouse, Priceline Pharmacy, Terry White Chemmart, Pharmacy 4 Less) or Google Shopping. A search term was typed in the search box of these websites. The terms used were categories of consumer products considered likely to contain antimicrobial biocides and to be used frequently based on our knowledge and previous research [4, 5], including personal hygiene and care products: ‘hand sanitizers’, ‘eye drops’, ‘throat lozenges’, ‘mouthwash’, ‘soap’, ‘hand wash’, and ‘antiseptics’, and domestic cleaning products: ‘spray cleaning’, ‘toilet cleaning’, ‘wet wipes’, ‘laundry sanitizer’. Home maintenance products were excluded. Though ear drops were initially included in the search, this category was not analysed further as only five non-prescription products were found, all of which were acetic acid-based treatments for earwax buildup. The terms ‘disinfectants’ and ‘wet wipes’ were also searched, and included products used for personal or cleaning use.

A product listing page of several consumer products was shown upon submitting a search term, and the ‘product detail page’ for each item was accessed. The following information from the product detail page was recorded: the brand, claims of efficacy, antimicrobial ingredients and their respective concentrations. Ingredients were recorded even if their in-product concentration was not available, or if the product did not list an ‘active ingredient’ required for certain claims of antimicrobial activity. Note that some compounds (for example, lactic acid, menthol) can be added to products for various reasons other than their antimicrobial activity; for completeness such products were retained in the dataset even if the intended role of the ingredient was unclear. All ingredients with antimicrobial activity were recorded for each product.

To ensure that no duplicate products were sampled across different retail websites, the exclusion criteria included products with identical brands, ingredients and % concentrations. If a listed consumer product exists in multiple ‘flavours’, only one ‘flavour’ was sampled in this study.

### Review of biocide regulation under the Therapeutic Goods Act

Relevant regulations and guidelines were found through the Australian Therapeutic Goods Administration website (www.tga.gov.au), which is responsible for implementing the Therapeutic Goods Act. Where specific sections of, or amendments to, the Therapeutic Goods Act were reviewed the exact article is also cited in the text.

## Results

**Fig. 2.**
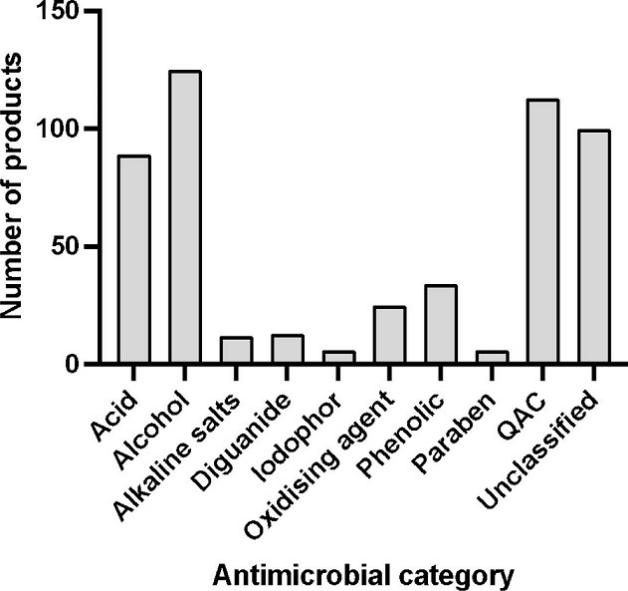
Overall prevalence of different categories of biocide across all products surveyed. Full data are available in the Supplementary Material. Where a product contained more than one additive in a specific category, the category was only counted once.

In total, 369 non-redundant biocide-containing consumer products were identified and classified into 13 product categories. These categories were found to contain 65 different antimicrobial ingredients, which were classified into eight groups – acids, alcohols, alkaline salts, diguanides, iodophors, oxidising agents, phenolics and QACs – based on chemical composition. The full dataset is provided as Supplementary Material. The most frequent biocides across the entire dataset were alcohols, QACs and acids (Fig. 2).

### Biocide additives in domestic cleaning and hygiene products

**Fig. 3.**
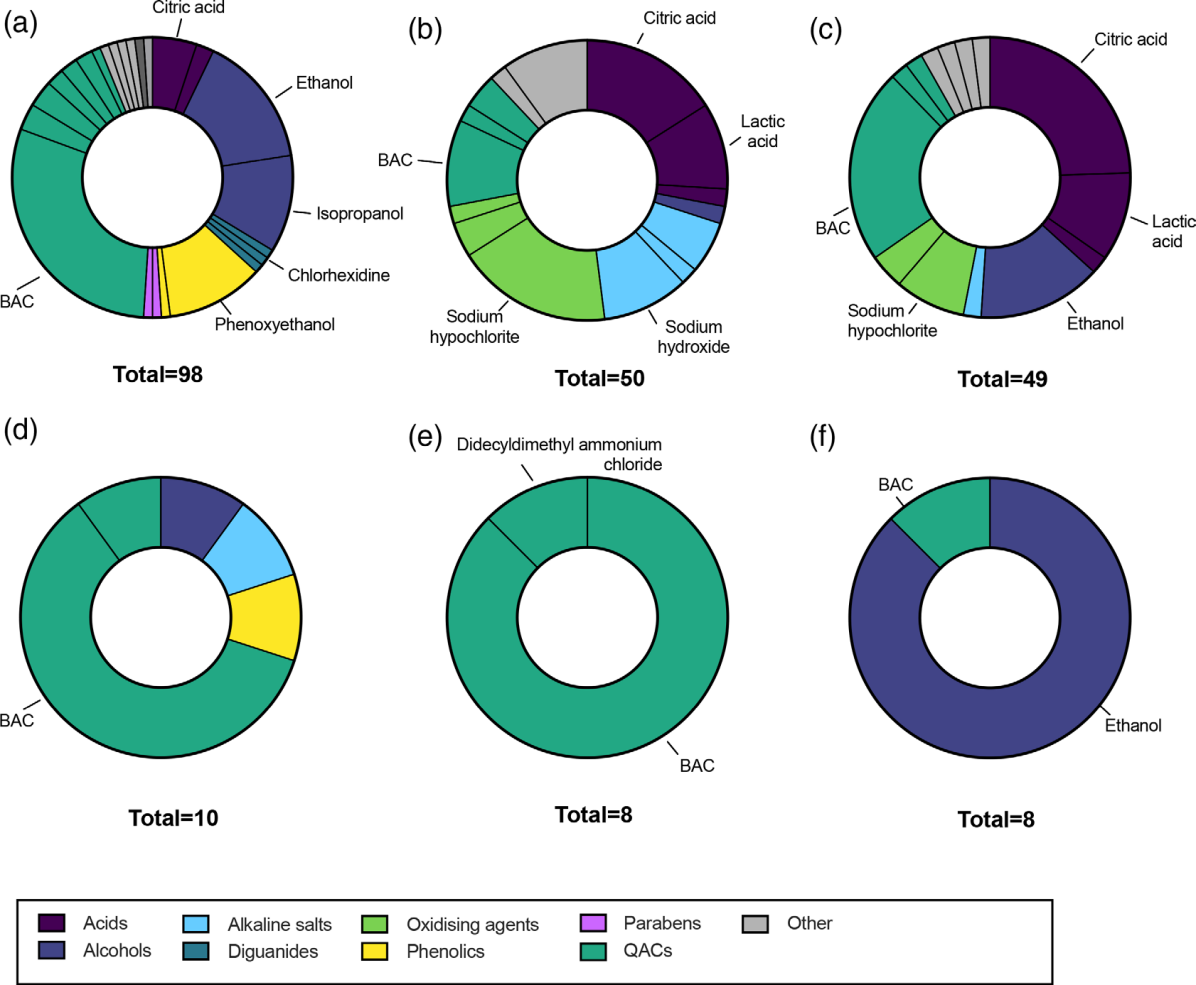
Antimicrobial additives found in domestic cleaning and hygiene products. (a) Antimicrobial wipes, (b) toilet cleaners, (c) spray cleaning products, (d) disinfectant liquids/sprays, (e) laundry sanitizers, (f) disinfectant aerosols. The total number of additives is greater than the number of products surveyed as >1 antimicrobial ingredient was recorded for many products. Individual segments represent specific ingredients, and these are coloured by category. Key antimicrobials are labelled. The complete dataset is provided in the Supplementary Material.

Domestic cleaning and hygiene products were surveyed in eight categories (Fig. 3): antimicrobial wipes, toilet cleaners, spray cleaning products, disinfectant liquids/sprays, laundry sanitizers and disinfectant aerosols. Antimicrobial wipes (Fig. 3a) were dominated by QACs and alcohols, one or both of which was present in 96 % of the products in this category. BAC and isopropanol were the most common antimicrobial ingredients in wipes, while phenoxyethanol was also widely used. Toilet cleaners (Fig. 3b) primarily used alkaline salts or oxidizing agents, such as sodium hypochlorite, though some products were based on QACs and weak acids. The most common additives in spray cleaning products (Fig. 3c) were weak acids (citric acid, lactic acid), followed by QACs.

The smaller product categories were found to be strongly associated with a particular biocide or biocide group. Disinfectant liquids or sprays (Fig. 3d) primarily contained QACs, and laundry sanitisers exclusively used QACs as the active ingredient. Aerosol disinfectant sprays (often marketed as deodorising) usually contained alcohol as the active ingredient.

### Antimicrobials used in personal hygiene products

**Fig. 4.**
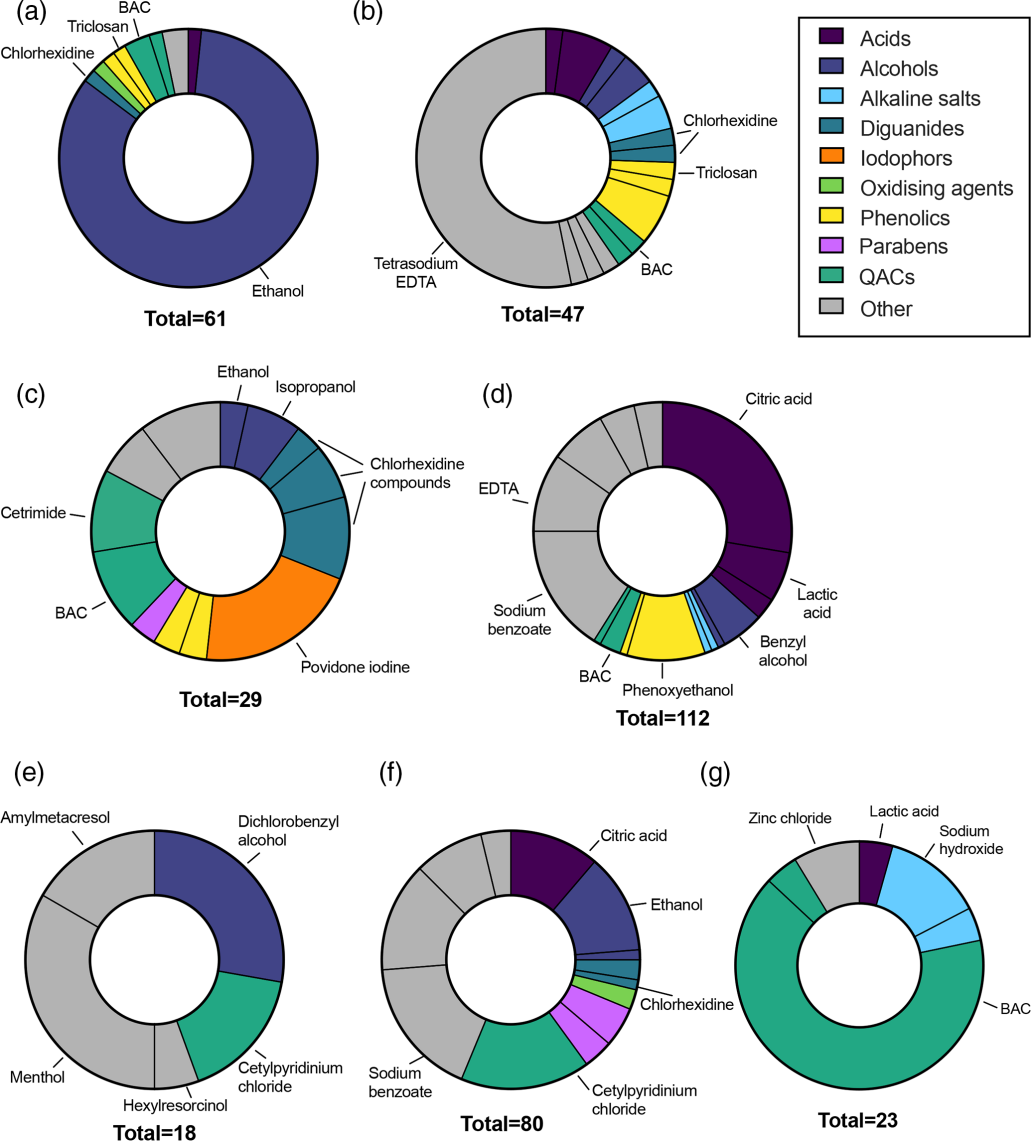
Antimicrobial additives found in personal hygiene products. (a) Hand sanitizers, (b) soaps, (c) skin antiseptics, (d) hand wash, (e) throat lozenges, (f) mouthwash, (g) eye drops. The total number of additives is greater than the number of products surveyed as >1 antimicrobial ingredient was recorded for many products. Individual segments represent specific ingredients, and these are coloured by category. Key antimicrobials are labelled. Full data is available in the Supplemental Material.

The use of antimicrobial additives in personal care products was more difficult to assign unambiguously due to the lack of standardization and frequency of products that do not make claims of antimicrobial activity. We surveyed products used primarily for personal hygiene – hand sanitizers, hand wash, soaps and skin antiseptics – as well as other products with other purposes but that frequently contain antimicrobial additives. The second category included mouthwash, throat lozenges and eye drops. Results are shown in Fig. 4.

Alcohols, particularly ethanol, were the most prevalent active ingredients in hand sanitizers (Fig. 4a). Triclosan and BAC were also detected in this product category, but only in a small proportion of products. Triclosan was also detected in soaps (Fig. 4b), although a large proportion of bar soaps did not contain specific antimicrobial additives apart from tetrasodium EDTA, which was likely included for formulation reasons and not its antimicrobial activity. Other biocides found in bar soaps included BAC and phenoxyethanol. Over-the-counter skin antiseptics (Fig. 4c) were likely to contain QACs, povidone iodine or diguanide antimicrobials like chlorhexidine. Common additives in handwashes (Fig. 4d) were citric acid or lactic acid, sodium benzoate and phenoxyethanol. Only a small proportion of handwash products contained other biocides, with 10 % of these products including a QAC. The majority of the antimicrobial activity of these products is presumed to come from the detergent itself.

Throat lozenges (Fig. 4e) included the antiseptic dichlorobenzyl alcohol (often combined with amylmetacresol), menthol, or the QAC cetylpyridinium chloride. The most frequently used antimicrobials in mouthwash products (Fig. 4f) included alcohols, QAC cetylpyridinium chloride, chlorhexidine and hydrogen peroxide. Finally, 80 % of eye drops (Fig. 4g) included a QAC (usually BAC) as a preservative, though zinc chloride was also used.

### Common biocide concentrations in consumer product categories

Not only is the identity of the biocidal active ingredient important, but its concentration is key to determining efficacy and eventual likelihood of selecting for AMR. Concentrations could only be determined on a small number of product categories with active ingredient listings. BAC showed a broad range of in-use concentrations ranging from 0.005–0.01 % in eye drops, to 0.1–1.5 % in cleaning products and surface disinfectants (spray cleaners, toilet cleaners, wipes and disinfectant sprays/liquids) to 7 % in laundry sanitizers. Alcohols were used at standard concentrations of 70–80 % v/v in hand sanitizers and antimicrobial wipes. Biocide concentrations were never specified on mouthwash, hand wash or soap products. This array of concentrations reflect the different reasons for adding the biocide, be it as an ‘active ingredient’, simply as part of the formulation, or as a preservative, and add a further layer of complexity for consumers and regulators. The vast majority of products where biocide concentrations were stated included these agents at high levels in excess of that required to kill microbes, meaning that exposure to sub-MIC levels most likely to drive microbial adaptation is unlikely to occur during correct product use, but may occur if there is environmental accumulation or incorrect use (for example, use of expired product). An exception is BAC in eye drops where concentrations were much lower, with the lower limit in the same range as concentrations shown to drive AMR *in vitro* [16].

### Biocidal claims in consumer products

Around 70 % of the consumer products, which include hand sanitizers, toilet cleaning products, disinfectant aerosols, antibacterial wet wipes, throat lozenges, laundry sanitizers, spray disinfectants and cleaning products, made some claim of antimicrobial efficacy. Of those products, the most common claim was ‘kills 99.9 % of germs’, which was present on 42 % of products. Other common biocidal claims were more specific, such as ‘hospital-’ or ‘household-grade disinfectant’ and ‘kills *E. coli*, *Salmonella* and COVID-19’. Approximately a third of investigated consumer products, which include most or all eye drops, mouthwashes, soaps and hand washes, did not make any claims of antimicrobial activity. In many cases the same active ingredients were present in products with and without claims of antimicrobial efficacy. The frequency of different types of claims is determined in part by how they are regulated in Australia, which will be discussed below.

### Australian legislation covering biocide usage and claims

Antimicrobial products for personal use are regulated as either therapeutic goods or industrial chemicals depending on the claims made and the intended use. While a comprehensive discussion of the current regulation in Australia is outside the scope of this work, some key points relevant to the inclusion of specific ingredients, and labelling of antimicrobial products, will be highlighted here.

The *Therapeutic Goods Act* of Australia (TGA) regulates medicines, medical devices, biologicals and other therapeutics, such as sterilants and disinfectants [31]. Products can fall within the remit of the TGA either because of specific ingredients included [listed under 52D(2)(b) of the TGA, also called the *Poisons Standard*], or because of their intended use and associated claims.

Of the domestic cleaning and hygiene products surveyed, many fall under the *Therapeutic goods (Standards for Disinfectants and Sanitary Products) Order 2019*; this states that all products with ‘specific’ claims of antimicrobial activity must pass a series of microbiological tests, which depend on the claims made. For instance, products that are ‘household-grade’ are tested against *E. coli* and *S. aureus*, whilst products with stronger claims like ‘hospital-grade’ must also use *P. aeruginosa* and *Proteus vulgaris* [32]. ‘Specific’ claims include activity against named micro-organisms, claims of activity against viruses, spores or fungi, or the terms ‘household-grade disinfectant’ or ‘hospital-grade disinfectant’. Products where the antimicrobial claims only extend to general activity against bacteria, for example, ‘kills 99.9 % of bacteria’, are excluded from the TGA under the *Therapeutic Goods (Excluded Goods) Determination 2018*, as this is not considered a specific claim. A summary of the testing required for antimicrobial products, and which products this rule applies to, is shown in Fig. S1.

Several of the personal care product categories surveyed are regulated as medicines under the TGA, including antiseptics, throat lozenges and eye drops; these are stringently controlled and require individual product registration. Hand sanitizers do not require individual registration, but are regulated by the TGA with specific guidelines for the ingredients permitted and their active concentrations, based on WHO recommendations [33]. Soaps, mouthwashes, antimicrobial skin wipes and hand wash products are regulated as industrial chemicals by default, unless they contain ingredients covered under the Poisons Standard, or make ‘specific’ claims of antimicrobial efficacy or to treat a particular disease.

The most frequently used biocide ingredients we identified are not covered in the Poisons Standard except at very high concentrations. For example, ethanol and isopropanol (at any concentration), chlorhexidine (at concentrations below 1%), QACs (at <5 %) and triclosan (at <0.3 %) are not restricted.

In addition to the TGA, products containing antimicrobial biocides can be regulated by other legislation including workplace safety law, consumer law and state/territory-specific laws. To our knowledge, no current legislation in Australia covering direct-to-consumer products explicitly addresses the potential for microbial adaptation to biocides, or for the potential for biocides to co-select for AMR.

## Discussion

Antimicrobial biocides are used very broadly in products available to the general public, but their usage in Australia was previously unknown. This research addresses this limitation by surveying biocides present across several different categories of consumer products, both in frequency and common concentrations. As the aim of the study was to survey the overall landscape of antimicrobial products that ordinary consumers might buy, we included both personal care and cleaning/domestic hygiene products in our study. In addition, this study reviewed the most common biocidal claims and the Australian legislation that regulates them. Such information is needed as a start point for further research, education and legislation aimed at improving biocide use.

We identified many different antimicrobial agents present in the Australian market. Some have additional functions as surfactants, descaling agents or in pH balancing, whilst others are used primarily to kill microbes. A limitation of this study is that in product categories that do not require ‘active ingredient’ listings, it was not possible to determine whether a potential biocide was present at a concentration sufficient to kill microbes. A further limitation is that we did not have access to sales volume information for any of the products, which would further inform future risk mitigation efforts. Nevertheless, the biocides detected in this study were similar to those reported in a survey that examines disinfectants and washing and cleaning products [4]. Alcohols, QACs (particularly BAC) and acids were the most common biocides across all products in our survey. In particular, QACs were frequently used in many different product categories in both household and personal care products.

Many factors determine whether antimicrobial biocides are used in consumer products and, if so, which ones and at what concentrations. From the perspective of microbial adaptation and selection for cross-resistance to antibiotics, some biocides are more problematic than others. Alcohols are one of the biocides of least concern [34] in domestic settings, given their rapid evaporation and lack of persistence in the environment; ethanol has also been reported to not select for cross-resistance to AMR [16]. Similarly, lactic or citric acid have low environmental persistence and low aquatic toxicity, and citric acid is now characterized as a biocide of least concern within the EU [35]. Reassuringly, these ‘safer’ biocides were widely used: ethanol (labelled as ethanol, alcohol or alcohol denat.) was the most prevalent biocide in our study, and dominated product categories including hand sanitizers, surface disinfectants and antimicrobial wipes, while citric acid was the third most prevalent biocide and was dominant in spray cleaning products and hand wash or soaps (Figs 3 and 4). Of more concern is the broad use of QACs, which have very well-documented links to AMR, moderate persistence, and are also released into the environment at concentrations relevant to microbial development of AMR [15, 16, 18]. BAC was the second most prevalent biocide identified in this work; this molecule was present in 22 % of products in our study across all categories, while 31 % of products contained either BAC or a different QAC. Widespread use of this biocide may be, in part, because it was once thought that QAC resistance and cross-resistance could not develop as they act on multiple cellular targets [14]. Nevertheless, further use of these biocides is predicted to increase the emergence of AMR bacteria, and there are additional concerns about its toxicity [14, 36]. Another concerning finding was that Triclosan was present in a few products, despite triclosan being banned in the USA and EU in many products due to its potential to worsen the development of AMR [17, 27, 28].

Many countries restrict antimicrobial biocide usage in order to prevent unwanted follow-on effects on AMR. In the USA, use of 19 biocides in household soap products was banned due to concerns about impacts on AMR pathogens and the environment, and a lack of evidence for improved efficacy [25, 26]. In the EU, the possibility of antimicrobial resistance due to biocides was considered as early as 2009 and resulted in recommendations that testing be developed and regulations put in place regarding cross-resistance [37]. From 2012 in the EU, biocide-containing products must be registered for use under regulations that require risk assessment regarding the development of resistance and cross-resistance in the target organisms [29]. Though this initially applied to ‘new’ compounds, over time the evidence accumulating against existing chemicals has led to revisions of their allowable concentrations and use, for example, BAC is no longer approved in products such as consumer hand and body wash antiseptics. We were encouraged to see that problematic biocides (e.g. BAC, chlorhexidine, triclosan) were found in only a handful of hand wash and soap products in our survey, and speculate that this is a flow-on effect from restriction of these additives overseas.

As detailed above, in Australia, antimicrobial biocides in consumer products are primarily regulated through the Therapeutic Goods Act, which considers toxicity and efficacy but does not explicitly address resistance and cross-resistance to antibiotics. Efficacy is well-regulated for some product categories but not others. Surface disinfectants are subject to mandatory testing under the TGA, broadly in line with what is used in the USA [38], while antiseptics, eye drops and throat lozenges are regulated relatively stringently as over-the-counter medicines. However other products including mouthwash, spray cleaning products, hand wash, soaps and antimicrobial wipes are not regulated by the TGA unless they make ‘specific’ claims of antimicrobial activity. Since claims like ‘kills 99.9 % of bacteria’ are not considered ‘specific’, this means that a high proportion of biocide-containing products, which people use specifically to protect themselves from germs, are exempt from TGA regulation.

The lack of uniformity in labelling makes the issue of biocides difficult for consumers to navigate. Many products with no antimicrobial claims still contained biocides such as BAC, while others claiming to ‘kill 99.9 % of germs’ contained no biocides or compounds of less concern such as weak acids. It is unlikely that the general public, who have a low level of understanding of AMR and disinfection in general [39, 40], would perceive a difference between the antimicrobial claims that are regulated (e.g. ‘household-grade disinfectant’, ‘kills *S. aureus*’) and those that are not, e.g. ‘kills 99.9 % of bacteria’. Overall the inconsistent labelling and regulation of antimicrobial products, and the broad range of chemicals used, means that consumer education alone will not prevent damaging biocides from being used unnecessarily and potentially contributing to the AMR crisis.

## Conclusions and future directions

Just as Australia undertakes stringent stewardship for antibiotics, with these medicines only available under prescription, we suggest regulations will prove more effective than education. The current regulatory framework for antimicrobial biocides in Australia results in inconsistent restrictions and requirements across very similar products, and a variety of antimicrobial claims that are difficult for consumers to understand. Relieving consumers of the responsibility of making these choices via regulation is the quickest and simplest way to reduce biocide use, and subsequent increases in AMR, in Australia. We suggest that adopting the ethos of the EU regulatory scaffold, whereby compounds need to be shown to not have adverse effects on AMR to be included in consumer products, is the preferred approach in order to avoid substitution of restricted chemicals for very similar non-restricted ones. We also suggest that a regulatory framework allowing control of biocide use across all categories of products will be more effective than the current situation where similar products are subject to very different levels of scrutiny.

This work also identifies specific product categories to prioritise in future research and regulation efforts. A high proportion of spray cleaning products, antimicrobial wipes and mouthwashes contained AMR-associated biocides (e.g. QACs), while these were less common in handwashes, soaps and hand sanitizers. The infrequency of problematic additives in soaps and handwash products likely stems from their regulation overseas, and is an encouraging sign that the unnecessary use of biocides can be addressed. Further work should examine biocides used in industrial and agricultural as well as domestic settings, in conjunction with information on usage levels, to gain a complete picture of how antimicrobial biocides are used in Australia.

## Supplementary Data

Supplementary material 1

Supplementary material 2
