# Supplementary material for: Cleaning up our disinfectants: usage of antimicrobial biocides in direct-to-consumer products in Australia
Source: Access Microbiol. 2024 Feb 14;6(2):000714.v3. doi: 10.1099/acmi.0.000714.v3 (PMC10928397; doi:10.1099/acmi.0.000714.v3)
Supplement: Supplementary material 1 [file acmi-6-714.v3-s001.pdf]

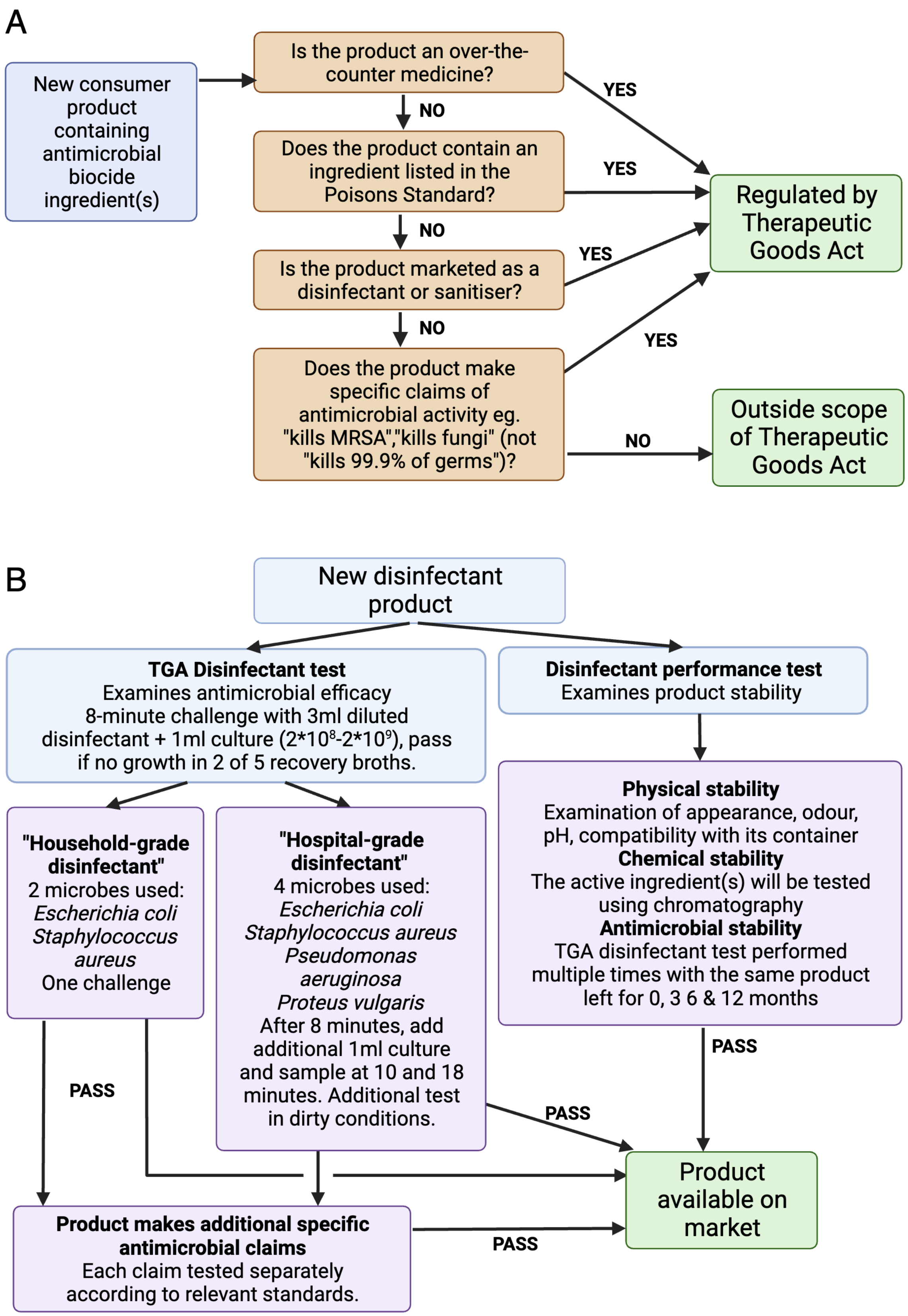

Supplementary Figure 1. Overview of TGA regulation of antimicrobial biocide-containing products. (A) Outline of products falling within, or excluded from, TGA remit. (B) TGA disinfectant testing. Note that the TGA provides a list of approved disinfectant formulations for which new testing is not required.
